# Academic competition-based learning cultivates scientific literacy to promote professional competitiveness in medical undergraduates

**DOI:** 10.3389/fpubh.2025.1590832

**Published:** 2025-07-03

**Authors:** Yan Ruan, Junlei Zhang, Jiali Wang, Rui Jian, Feng Mei, Hongli Li, Yun Zhang, Qiwen Hu, Lan Xiao, Yi Yang, Ming Li, Jiaxiang Xiong, Yanping Tian

**Affiliations:** ^1^Department of Histology and Embryology, College of Basic Medical Science, Army Medical University, Chongqing, China; ^2^Experimental Center of Basic Medicine, College of Basic Medical Science, Army Medical University, Chongqing, China; ^3^Department of Foreign Languages, College of Basic Medical Science, Army Medical University, Chongqing, China; ^4^Department of Microbiology, College of Basic Medical Sciences, Army Medical University, Chongqing, China

**Keywords:** academic competition, scientific literacy, professional competitiveness, undergraduate education, active learning, critical thinking

## Abstract

**Background:**

Contemporary healthcare requires medical professionals with advanced scientific literacy. Current undergraduate medical curricula may not consistently develop this critical skillset. This study evaluates the effectiveness and challenges of an academic competition-based learning (ACBL) for enhancing scientific literacy in medical undergraduates.

**Methods:**

The International Genetically Engineered Machine (iGEM) Competition based program was developed using a two-round modified Delphi study. 30 students participated in an iGEM-based academic competition during 18 months. Scientific literacy domains were assessed through validated questionnaires during a five-year follow-up period.

**Results:**

iGEM participants demonstrated significantly greater improvement in literature review, experimental design, technical execution, presentation skills, and research management compared to controls (*p* < 0.01). Significant gains were observed in scientific knowledge acquisition and scientific reasoning (*p* < 0.01). Scores for active learning, critical thinking, and collaborative communication were significantly higher in the iGEM group (*p* < 0.05). Participants identified laboratory resources, space, equipment and funding as primary implementation constraints.

**Conclusion:**

ACBL is an innovative and effective strategies to develop students’ scientific literacy for professional competitiveness, which highlights the potential of ACBL as a transformative approach in medical education.

## 1 Introduction

Global healthcare demands are shifting from disease-centered models toward lifelong health maintenance, requiring professionals with enhanced scientific literacy to deliver high-quality care and drive innovation (1, 2). Scientific literacy is the ability to creatively utilize appropriate evidence-based scientific knowledge and skills in solving challenging yet meaningful scientific problems as well as making responsible scientific decisions, of which problem-solving, critical thinking (CT), communication and the ability to interpret data are four core components (2–4).

Cultivation of scientific literacy is a systematic work, which needs practice and accumulation for a long time (5, 6). Research experience during undergraduate medical education is an opportunity to develop scientific literacy for undergraduates, which can be conducive to career developments of participants (7–9). Research experiences include Undergraduate Research Experiences (UREs) and Course-based Undergraduate Research Experiences (CUREs) (10, 11). UREs feature individual students in faculty laboratories and provide the opportunity, but the most common problem is their treatment as cheap labor for repetitive and time-consuming lab work resulting in insufficient participation. CUREs is a course-based training program and open to most students. Although CUREs offer broader access, their effectiveness may be constrained by short durations and high student-to-faculty ratios (10, 12–15).

Academic competitions are increasingly integrated into educational strategies to motivate students and enhance learning (16). Academic competition-based learning (ACBL) is a process of active acquiring knowledge within a competitive setting, which stimulates students’ problem-solving abilities, improves learning efficiency, and enhances confidence as students-centered learning model (16, 17). More and more undergraduates are improving their scientific literacy by participating in various academic competitions (18–20). ACBL has been successfully implemented in numerous fields, including engineering, computer programing and information systems, as well as physics and biology (16). However, ACBL’s application for cultivating scientific literacy in medical undergraduates remains underexplored.

The purpose of this study is to evaluate the effects of ACBL on scientific literacy for medical undergraduates. The results showed that ACBL engaged student’s innovation ability, sparks active learning and cultivate critical thinking and collaboration and communication skills. In conclusion, ACBL may be an effective learning strategy for medical undergraduates in enhancing scientific literacy to promote professional competitiveness, which highlights the potential of ACBL as a transformative approach in medical education.

## 2 Methods

### 2.1 Participants and study design

**Figure 1:**
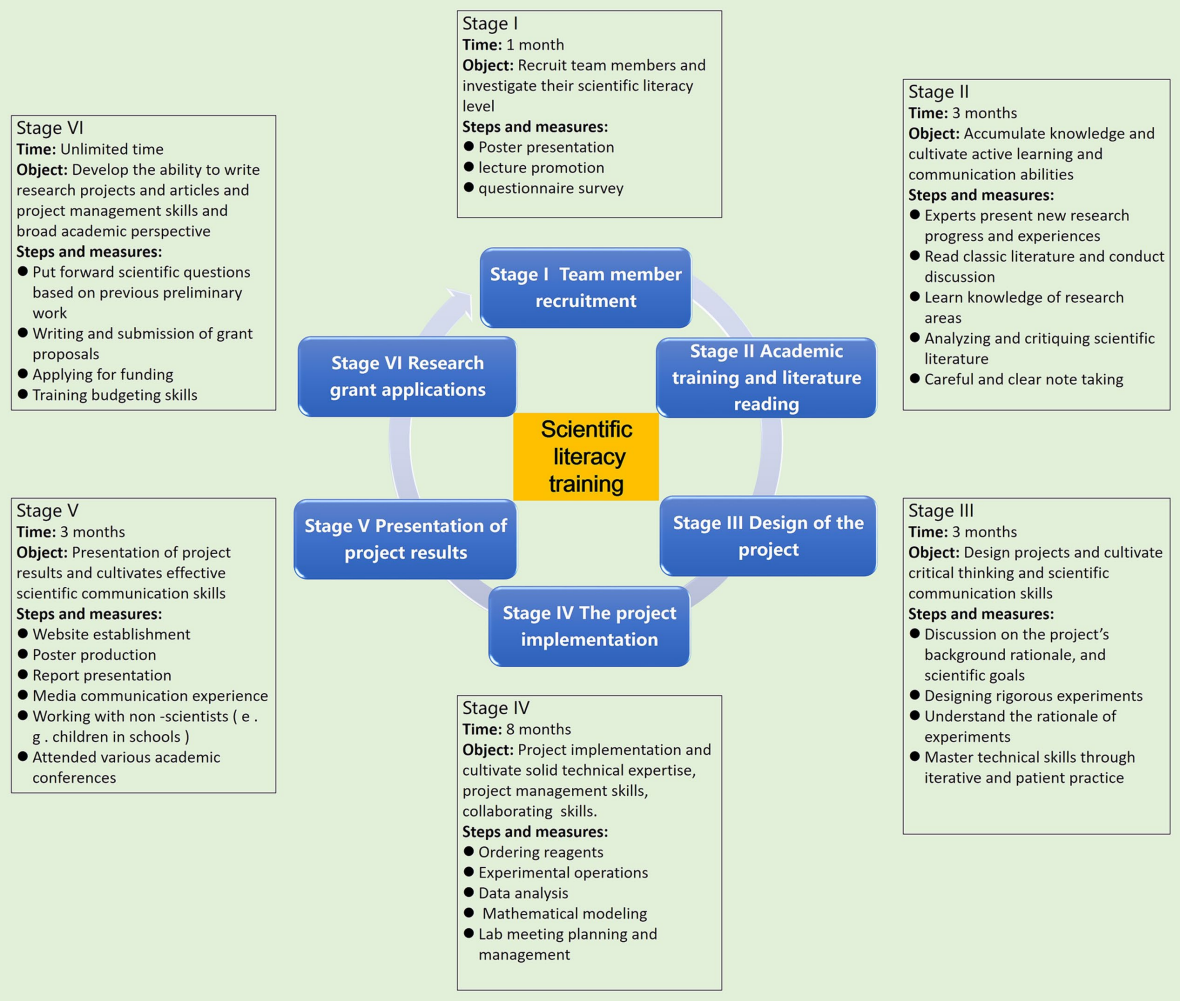
Schematic flowchart showing the design of the academic competition-based leaning program. Flowchart illustrates six stages of the academic competition-based leaning program: recruitment, academic training, project design, implementation, presentation, and research grant applications. Each stage specifies timeframe, objective, and step. Central focus is on fostering scientific literacy.

The ACBL program was developed around the International Genetically Engineered Machine (iGEM) competition. The iGEM competition is a series of competitions that develop and undertake synthetic biology research projects, which began in 2003 at MIT, MA, USA and developed into an international academic competition in 2005.^1^ The iGEM aims to encourage interdisciplinary collaboration and innovation among undergraduate participants (21–23). The conceptual framework of the ACBL program based on the iGEM competition is illustrated in Figure 1. All aspects of the research process were conducted over a three-semester period or more.

#### 2.1.1 Stage 1. Team member recruitment

Eighteen months prior to iGEM competition, 30 undergraduates were recruited from Third Military Medical University (Chongqing China). Faculty selection occurred after voluntary registration of students based strictly on competition guidelines and ethical principles.

#### 2.1.2 Stage 2. Academic training and scientific literature reading

The research content and activity mode of ACBL were constructed by a two-round modified Delphi study (24). After the recruitment of team members completed, experts in related fields were invited to instruct new research progress and activity experiences including literature searching, experimental method, and paper writing (Supplementary Figure S1). Team based learning (TBL) provides an active, structured form of small group learning. Each group read classic literature and then conducts regular discussion and to complete a literature review. Three types of literature are provided: (1) research articles closely related to the ongoing project; (2) balanced and comprehensive reviews with a broad scope covering the relevant field; and (3) classic landmark papers with brilliant experimental designs (15).

#### 2.1.3 Stage 3. Experimental design of the project

On the basis of previous learning, the team members conducted experimental design for the project and held weekly project discussions in the form of regular meetings to improve project designs including the project’s background, rationale, and scientific goals. Tutors provide guidance on scientific research ideas and design. The students also understand the rationale of experiments and master technical skills through iterative and patient practice (Supplementary Figure S1).

#### 2.1.4 Stage 4. Implementation of the project

Team members are assigned experimental operation group, data analysis group and mathematical modeling group based on their own strengths and complete their tasks. Tutors provide guidance on implement of their plans. Weekly lab meetings provide a platform for students to practice oral communication skills and present their discoveries (Supplementary Figure S1). The participating team members also need to engage in social activities, including soliciting public feedback on the topic and listening to expert opinions.

#### 2.1.5 Stage 5. Presentation of the project

Teams prepared competition deliverables, including project websites, explanatory videos, posters, and oral presentations (Supplementary Figure S1). The students also actively attended various academic conferences and presented their work. Finally, students present their achievements on the annual meeting in the final of the iGEM competition in Boston (USA).

#### 2.1.6 Stage 6. Research funding application and management

After the completion of the academic competition, the students were voluntary to train how to put forward scientific questions based on previous work, write and submit research funding application, and how the funding would allow them to conduct experiments to address the underlying questions.

#### 2.1.7 Control group

A control group (CTL, *n* = 30) comprised same-grade, same-major peers who confirmed no participation in academic competitions during the study period. Table 1 shows the basic characteristics of the two groups (iGEM group and CTL group). All students have completed the compulsory course learning, and there was no significant difference in Grade Point Averages (GPAs) between the groups. All participants completed scientific literacy assessments before the training activities.

**Table 1:** Demographic characteristics of the studied population by groups.

Groups	iGEM group number (%)	CTL group number (%)
Total number of students	30 (100.0)	30 (100.0)
Mean age (years) mean (± SD)	21.4 (0.84)	21.8 (1.13)
Gender		
Male	18 (60)	17 (56.7)
Female	12 (40)	13 (43.3)
Major		
Clinical medicine	20	20
Preventive medicine	6	5
Others	4	5
Program		
4-year program	3	3
5-year program	23	23
8-year program	4	4
Grade		
Second year	4	4
Third year	19	19
Fourth year	7	7

CTL group, Control group; iGEM group, iGEM competition group; SD, standard deviation.

### 2.2 Data of achievements of students participated in iGEM competition

After the iGEM competition, published research papers and innovative entrepreneurial training plan program or other research grants received by students were collected. Moreover, the enrolment rate of postgraduate education was counted for undergraduates participated in iGEM competition.

### 2.3 Questionnaires for students’ satisfaction with the scientific research experience and challenges in implementation of ACBL

All participants are invited to complete the pre-training and post-training questionnaire, respectively, by a quantitative empirical method (five-year follow-up). The questionnaire items covered the view on the process of scientific research (5 items). The second section reflected the subjective influence of the ACBL program including active learning (9 items), critical thinking (14 items) and collaboration and communication (9 items) (2, 25). Moreover, challenges in implementation of ACBL were also analyzed by a questionnaire survey. All items used a 5-point Likert scale (1 = Strongly Disagree, 5 = Strongly Agree).

### 2.4 Statistical analyses

The data were analyzed using the Statistical Package for Social Sciences software (SPSS, version 26, IBM Corp.). Descriptive statistics were used to present an overview of the data. Wilcoxon rank sum test was performed within both groups. The statistical analysis of the satisfaction questionnaire was performed using the average score of all items in each construct. The quantitative content analysis was performed to manage qualitative data from the open-ended questions. *p*-values of less than 0.05 were considered significant.

## 3 Results

### 3.1 Participation of iGEM and CTL groups

**Figure 2:**
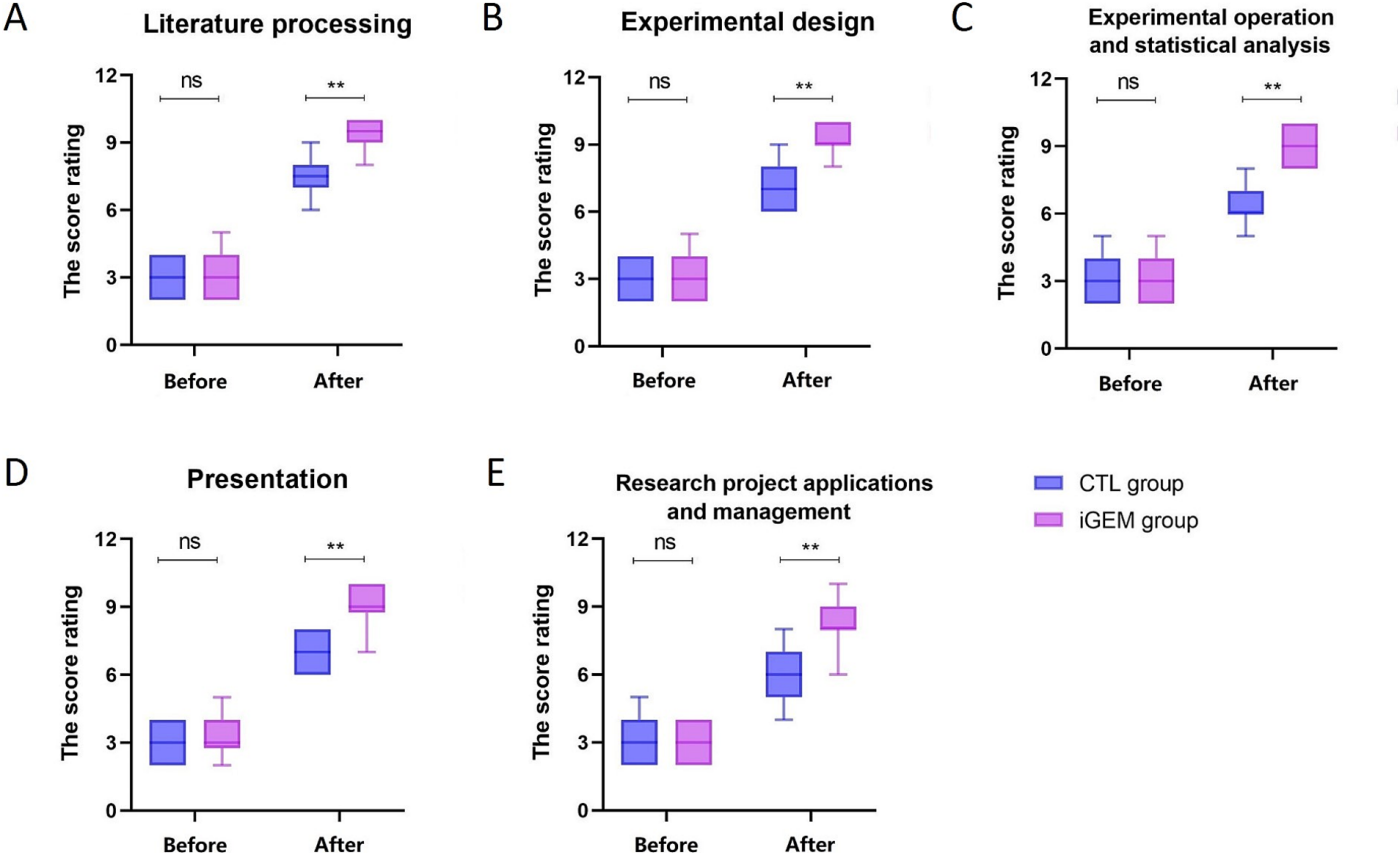
Comparison of the scores of scientific research ability before and after iGEM competition in the iGEM (purple) and CTL (blue) groups. Five box plots labeled **(A–E)** compare score ratings for two groups. Significant improvements after the intervention for both groups in the following areas: **(A)** Literature processing, **(B)** Experimental design, **(C)** Experimental operation and statistical analysis, **(D)** Presentation, and **(E)** Research project applications and management. The improvements are statistically significant (noted by “**”), except for the initial comparison labeled as “ns” (not significant).

A total of 60 students participated in this study. Table 1 compares the basic characteristics of the iGEM and CTL groups. There were no significant differences between the two groups in terms of grades, gender, or age (*p* > 0.05). All students and tutors were participated in the survey, with a response rate of 100% in both groups. The results showed there is no significant difference in scientific literacy between the two groups before the training (Figure 2).

### 3.2 Results of iGEM competitions and achievements of students after iGEM competition

Three teams participated in iGEM competitions from 2016 to 2019, winning two Gold medals and one Silver medal. Epithelial mesenchymal transition (EMT) refers to the process by which epithelial cells acquire characteristics of mesenchymal cell, which is crucial for embryogenesis, wound healing and malignant progression (26). In 2019, we constructed a visualized cellular EMT model and simulated the dynamic changes of EMT by establishing a mathematical model which is of great significance for the understanding of EMT (Supplementary Figure S2A). Based on reasonable design and innovative discoveries, our project won gold award and nominated for the best basic research award (Supplementary Figure S2B).

In the evaluation of achievements, the students participated in the iGEM competition published 19 research papers in academic journals showing students being first author and co-first author in 10 papers, other co-author in 9 papers (0.63 articles per person, Supplementary Table S1). In the CTL group, 2 students published articles as first authors (co-first author) and 2 students published articles as co-authors. Another encouraging finding is that 30% (9/30) of students received college students’ innovative entrepreneurial training plan programs or other research grants compared with 10% of students in the CTL group. These achievements stimulated their interests and pursuits of medicine and life sciences, prompting them to further their studies. Students participating in iGEM and others trained based ACBL also won national academic competitions in their subsequent study periods, such as the National Forum for undergraduate on Innovation research and Experimental Design Competition and National Undergraduate Life Science Competition (Supplementary Table S2). We also found that 80% (24/30) of students were enrolled postgraduate education, but 53.5% in CTL group. These results suggest that participation in the iGEM competition significantly enhances students’ scientific literacy.

### 3.3 Comparison of the scientific research ability between the iGEM and CTL groups

Scientific research activities are a long-term and complex process, including literature processing, experimental design, experimental operation and statistical analysis, presentation and research grant applications and management. Our results showed that students who participated in the iGEM competition gained a greater understanding and appreciation of the research process (Figure 2). Our results showed that students in iGEM group had a higher literature processing level (9.33 ± 0.76) than CTL group (7.57 ± 0.82, *P* < 0.01). Students demonstrated increased experimental design and experimental skills (*P* < 0.01). They acquired better capability in paper writing and presentation (9.20 ± 0.89 vs. 6.97 ± 0.85, *P* < 0.01). Students who participate in iGEM competition are equipped with better evaluation of research grant applications and management than those who do not (8.33 ± 0.99 vs. 5.83 ± 1.02, *P* < 0.01).

### 3.4 Analysis of questionnaire of iGEM and CTL groups on active learning, critical thinking and collaboration and communication skills

Students rated the project positively, from good to very good on active learning, learning efficiency, and integration of knowledge (Table 2). Most of students reported they had enjoyed active learning (*n* = 28, 93.3%). They felt that the greatest contributions of ACBL program lies in the increased medical knowledge and scientism in the research process. Students improved time management (*n* = 27, 90%) and increased ability to work autonomously (*n* = 26, 86.7%). All of students (*n* = 30, 100%) also reported the training increased research knowledge and an enthusiasm for scientific research. We have assessed whether or not the medical students’ participation in scientific researches affected their study of medical curriculum. The results showed instead of affecting the study of the medical curriculum, academic competition enables the reflection and integration of learning.

**Table 2:** Questionnaire results about active learning rating with the 5-point Likert scale in the iGEM and CTL groups.

Items	CTL group	iGEM group	Wilcoxon statistic	*P*-value
1. The project help me learn actively new knowledge	3.80 ± 0.66	4.53 ± 0.63	206	0.0001
2. The project help me work and learn independently	4.13 ± 0.68	4.47 ± 0.73	327	0.0484
3. The project help me acquire time management skills	4.27 ± 0.64	4.53 ± 0.68	342	0.0775
4. The project improved my learning efficiency	4.10 ± 0.66	4.63 ± 0.49	257.5	0.0016
5. The project enhanced my learning experience and interest in the course	4.00 ± 0.69	4.67 ± 0.48	220	0.0002
6. The project increased my medical knowledge in the course	4.30 ± 0.53	4.77 ± 0.43	251.5	0.0007
7. The project enhanced knowledge of research area	3.80 ± 0.61	4.80 ± 0.41	108	<0.0001
8. The project are conducive to the integration of learned knowledge	3.97 ± 0.67	4.63 ± 0.49	216	0.0001
9. The project does not affect my course learning	4.63 ± 0.49	4.80 ± 0.41	375	0.1582

CTL group, Control group; iGEM group, iGEM competition group.

The growth in CT scores increased in both groups five-year follow-up, but students participating in ACBL increased the CT test score significantly more than those students who did not have this experience (Table 3). Research experience promoted them to think critically, improved skills of data analysis and project design, and enabled them to succeed in a lab environment (*P* < 0.01). Students (*n* = 30, 100%) also reported the project enabled them to conduct more researches in the future. Our results showed that ACBL improved CT for undergraduates.

**Table 3:** Questionnaire results about critical thinking rating with the 5-point Likert scale in the iGEM and CTL groups.

Items	CTL group	iGEM group	Wilcoxon statistic	*P*-value
1. I can formulate a clarity research hypothesis	3.60 ± 0.62	4.37 ± 0.61	194	<0.0001
2. I can use the appropriate tools, materials, and equipment to conduct research	3.30 ± 0.60	4.53 ± 0.51	77	<0.0001
3. I can determine the appropriate experimental methods to investigate research results	3.47 ± 0.57	4.60 ± 0.50	93	<0.0001
4. I can collect data of my research project	4.20 ± 0.61	4.67 ± 0.50	270	0.0028
5. I can determine statistical methods to analyze data	3.33 ± 0.66	4.53 ± 0.51	91	<0.0001
6. I can ask questions to clarify my understanding of my research project	3.60 ± 0.72	4.40 ± 0.67	204	0.0001
7. I can provide a clear statement of the conclusion	3.70 ± 0.65	4.56 ± 0.50	162	<0.0001
8. I can analyze the correlation between experimental results and conclusions	3.57 ± 0.73	4.47 ± 0.63	177	<0.0001
9. I can design and conduct a research project	3.00 ± 0.64	4.37 ± 0.61	75	<0.0001
10. I can accept suggestions to improve my research	4.37 ± 0.61	4.53 ± 0.51	391	0.3257
11. The project improved my critical thinking skills to solve problems	3.77 ± 0.68	4.57 ± 0.50	183.5	<0.0001
12. The project improved my reflective thinking	4.10 ± 0.71	4.53 ± 0.63	309	0.0229
13. The project improved my confidence	3.90 ± 0.66	4.63 ± 0.49	196	<0.0001
14. The project made me conduct more research in the future	4.27 ± 0.64	4.67 ± 0.48	300	0.0124

CTL group, Control group; iGEM group, iGEM competition group.

Our activities encourage undergraduates to collaborative learning and give them more opportunities to present their own discoveries. Participants felt the ACBL benefited them to improve collaboration and communication skills (Table 4). Students (*n* = 28, 93.3%) reported that the training had both increased their collaboration skills and their ability to communicate with staff. 96.6% of students (*n* = 29) reported increased overall confidence, with which students can be more actively involved in research and assist their peers in further learning. Encouragingly all students (*n* = 30, 100%) would definitely recommend our activities to fellows.

**Table 4:** Questionnaire results about collaboration and communication skills rating with the 5-point Likert scale in the iGEM and CTL groups.

Items	CTL group	iGEM group	Wilcoxon statistic	*P*-value
1. I can complete experiments collaborated with peers in lab	3.83 ± 0.65	4.67 ± 0.48	165	<0.0001
2. I can communicate with peers in lab	4.43 ± 0.63	4.80 ± 0.41	309	0.0126
3. I can communicate with tutors and PI	3.60 ± 0.62	4.50 ± 0.63	162	<0.0001
4. I can express my views in lab meeting	4.07 ± 0.69	4.63 ± 0.49	252	0.0012
5. I can present my results of my research at a research symposium	3.37 ± 0.61	4.57 ± 0.50	84.5	<0.0001
6. I am confidence talking to peers and tutors	4.00 ± 0.69	4.50 ± 0.63	277.5	0.0056
7. I can tailor my research communications for different audiences	4.23 ± 0.57	4.57 ± 0.50	317	0.0253
8. I can write research papers	3.57 ± 0.63	4.53 ± 0.57	141.5	<0.0001
9. I would recommend this training program to others	4.73 ± 0.45	4.87 ± 0.35	390	0.2043

CTL group, Control group; iGEM group, iGEM competition group.

### 3.5 Challenges in implementation of ACBL

**Figure 3:**
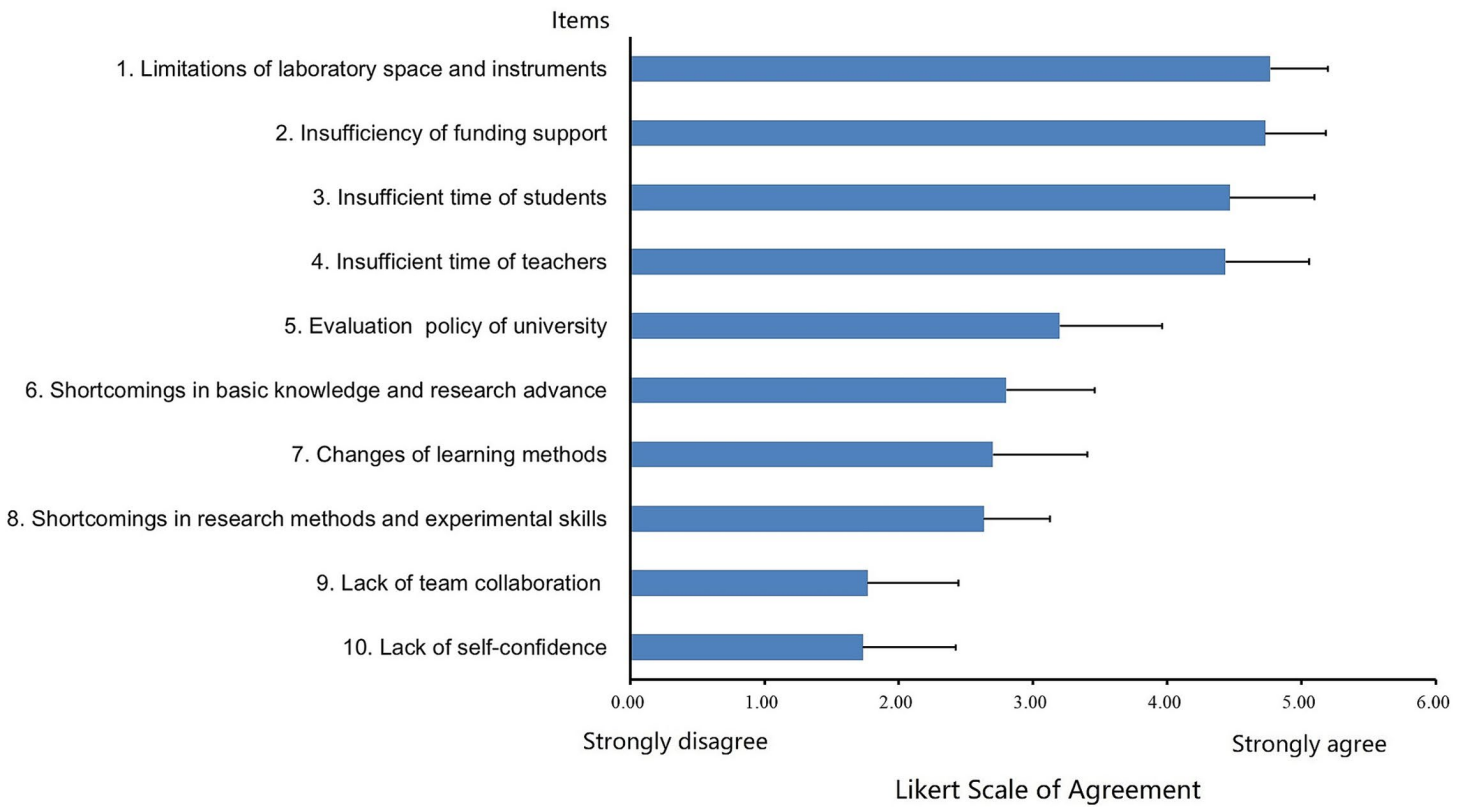
Factor analysis for challenges of implementation of ACBL from students and tutors participated in iGEM competitions. Bar chart shows Likert Scale of Agreement for various educational challenges. Key challenges include limitations of laboratory space, funding, and time for students and teachers. Bars range from strongly disagree to strongly agree, with variations in agreement levels.

Although ACBL improved scientific literacy for undergraduates, the implementation of academic competitions is accompanied by various difficulties and challenges, which are summarized in Figure 3. Our results of questionnaire survey showed that the biggest obstacles were limitations of laboratory space and instruments and insufficiency of funding support, which filed to provide enough training opportunities to facilitate undergraduate development. Insufficient time of students and teachers also constrained the implementation of academic competitions because both students and teachers need to invest a lot of time and energy during implementation of academic competitions.

## 4 Discussion

Enhancing scientific literacy is crucial for preparing medical professionals to navigate the complexity and rapid evolution of healthcare (2). Our university cultivates undergraduates with scientific literacy through series of academic competitions. In this study we analyzed scientific literacy of undergraduates participated in iGEM competition, who not only gained comprehensive research experience but also received professional academic guidance and interpersonal skills training. Our findings suggest that ACBL can be an effective approach for enhancing scientific literacy to promote professional competitiveness among medical undergraduates.

Opportunities for developing scientific literacy within compulsory courses can be limited, potentially impacting research awareness and methods among undergraduates in China (27). Experience of scientific research is the most effective way to cultivate the scientific literacy for medical students. As the study reported by Huang et al., research programs could help medical undergraduates build interest in scientific research and develop scientific thinking and basic research capacities (28). Academic competition is a key element in many educational approaches and is often adopted by educators in an effort to motivate and excite their students although opponents argue that academic competition causes an increase in student anxiety and divides their attention (16, 29). Academic competitions both strengthen student motivation for academic improvement and also engage students in relevant academic content. Participants reported enjoying the professor’s inclusion of competitive elements by choosing a competitive learning task that is high in energy and short in duration (16).

Recognizing the value of scientific literacy development, Chinese government has carried out a series of academic competitions, including National Clinical Skills Competition, National Undergraduate Life Science Competition and “Challenge Cup” National Undergraduate Extracurricular Academic Science and Technological Works Competition (Supplementary Table S2) (17–20). These programs often incorporate advanced pedagogical principles, shifting toward student-centered, project-based learning models designed to foster scientific literacy. The results of surveys suggested that these competition programs deliver a high-quality learning environment and improves learning outcomes compared to traditional work-integrated learning (17, 18). In summary, academic competitions are increasingly viewed as influential tools for medical education reform.

The iGEM competition exemplifies a high-impact, international platform mobilizing student creativity and initiative to develop scientific literacy (30). The students from internationally prestigious universities participate in the competition, including Stanford University, MIT, Harvard University, and Oxford University. Top universities in China including Peking University, Tsinghua University and Zhejiang University also participate in iGEM competitions (22). As a research-intensive university, our undergraduates have participated in three iGEM competitions winning two gold and one silver awards. Undergraduates participated in iGEM competitions excel at the research process. To accumulate knowledge related to the research field, the most effective way is the comprehension and analysis of literatures. Proposing and designing scientific research projects reflect students’ sensitivity to scientific research, which is also a way to apply their theoretical knowledge, innovative thinking, and comprehensive quality in scientific research (28, 31). We encourage undergraduates to read classic literatures and design experiments by themselves in our activities. Training of experimental techniques can greatly improve students’ scientific research skills. Medical undergraduates can better understand principles and procedures of experimental operations by involving themselves in experimental operations in the research training. They also were trained better capability in scientific writing and presentation, which is one of the most important abilities in research activities. Projects application and management are often missing from undergraduate training, mainly because their lab work is usually defined by individual experiments rather than a complete scientific project. Unlike other studies, we encourage students to participate in the application for research projects, forming an entire experience of research work, which is an important component of our ACBL. Our combined assessments indicate that both students and faculty benefit from these competitions, as students develop scientific literacy and basic research capacities and faculty also benefited from increased student participation and collaboration (32). Hence, our training program can help medical undergraduates build interest in scientific research and develop scientific literacy, which promote their professional competitiveness (33).

The dynamic nature of medicine necessitates a capacity for active, lifelong learning among healthcare professionals. Active learning is the foundation for lifelong learning for medical students (34). It is essential for early training on active learning methods due to Chinese students lack strong motivation for active learning. Therefore, cultivating active learning was a core objective of our ACBL program. We encourage students to actively learn to acquire new knowledge on account of academic contexts being highly competitive and complex. Participants reported effectively employing active learning strategies during literature review and analysis, creating a more immersive and impactful experience than traditional classrooms. ACBL also enhances student the reflection and integration of learning to promote long-term retention in learners (35). These skills they gained from research group are important in the medical profession and should be developed and nurtured.

Critical thinking (CT) is indispensable for clinicians, enabling sound decision-making, judgment, and inference in complex clinical situations (36). CT is a cognitive process to identify and analyze problems and seek and evaluate relevant information to reach an appropriate conclusion, which includes various skills including analysis, evaluation, inference, deductive reasoning, and inductive reasoning, which is a desirable skill for clinical professionals (37–39). The “World Federation for Medical Education” and Institute for International Medical Education (IIME) have introduced CT as one of the basic standards of medical education, which has thus become the fundamental skill for cultivating innovative talents (40, 41). A central goal of our training is to promote students to think critically. We argue that the key element for developing this ability is repeated practice in making decisions based on data, with feedback on those decisions. We provide many such opportunities for undergraduates in our competition activities. In research works, crucial decisions are to embrace, adjust, or discard a model based on the scientific evidence; or to devise a new experiment to answer the question (39, 42). We encourage undergraduates to fearlessly ask questions based on the experimental data until every puzzle in your mind is resolved, thereby laying a solid foundation for the formation of their own CT. Many students believe that our training program aids their CT skills as an excellent learning experience.

Effective teamwork and communication are essential in modern healthcare, extending beyond patient interactions to collaboration with colleagues and staff (43). Medical undergraduates need to develop teamwork and communication skills as they have to collaborate with other health professionals while attending to patients in the future works. The inherently interactive and collaborative nature of our ACBL program provided valuable training in communication and teamwork. In this study, different roles and scenarios were prepared to enhance students’ collaborative skills and consciousness. Team members undertake different tasks, which must collaborate with each other to complete complex competition procedures. Weekly lab meetings and various academic conferences provide a platform for students to practice presentation skills. We find that presentation in lab meetings is a remarkably effective way to improve communication skills for undergraduates. Regular lab meetings and academic conferences offered crucial platforms for practicing communication skills, significantly boosting confidence and performance (30, 35). These outcomes are also the ultimate aim of higher education, which intends to cultivate students’ competences in collaboration and communication instead of only focusing on learning outcomes.

As high-impact educational practices, ACBL provides excellent opportunities for medical undergraduates to improve scientific literacy, but it is important to recognize the difficulties and challenges associated with running such projects. Firstly, while academic competitions provide an abbreviated but in-depth exposure to the research process, only a small number of students participate in this process due to limitations in lab space and equipment. Importantly, teams require a significant amount of funding to participate in academic competition especially in international academic competitions, which includes registration fee and travel fee, and some associated with the project (research consumables and equipment). But many universities cannot provide sufficient funding to support these activities. Secondly, an increasing participation places a greater burden on faculty time and resources although research experiences provide greater benefits and gains for undergraduate. The chief difficulties are the time and energy of faculty required for project design and implementation, which must be balanced with the other responsibilities of the faculty who conduct both research and teaching obligations. Thus, medical undergraduates who wish to conduct research activities need to find professors that are willing and available to orient their projects (10, 15). Lastly, the academic competitions are usually implemented during extracurricular time. Undergraduates may not have enough time to run an entire project due to heavy learning tasks and other activities. Time scarcity was consistently reported as a major obstacle, echoing findings by Wan et al. that undergraduates often find research stressful due to workload pressures (44).

Providing intensive research experiences like iGEM to all undergraduates may not be feasible. However, offering such opportunities to selected students represents a valuable investment in future medical leadership (45). Students participating in academic competition have clearly identified that the competitions provided a range of experiences and skills that will benefit their undergraduate studies and future professional competitiveness. Proper training of undergraduates takes time and effort, but we believe that such time and effort are well worth spending and provide huge benefits consistent with opinions of Marla B. Feller (46). Moreover, fostering undergraduate with scientific literacy requires not only the effort of faculties and students themselves but also support from institutions fellowships or department funds specifically designated to support undergraduates training. College administrators should strive to create incentives for faculty members to collaborate with students and provide additional resources to improve scientific literacy for undergraduates.

## 5 Limitations of the study

There are some potential limitations of the current study. A general limitation of this study was that the number of participants was relatively small, and therefore not providing more information about the academic competition. Another limitation of this research is that it was conducted over only 4 years at one university. Additional research is needed to better understand the application of the ACBL over a longer time period and more universities.

## 6 Conclusion

Enhancing scientific literacy in medical undergraduates is crucial for individual career advancement and the overall quality of healthcare services. Our evidence suggests ACBL effectively engages students in novel scientific research performance, fostering significant improvements in scientific literacy, active learning, critical thinking, and collaboration and communication skills, which are key attributes for professional competitiveness. However, successful implementation requires substantial institutional commitment, including adequate laboratory resources, dedicated funding, and faculty support to overcome inherent challenges of time and resource constraints. In conclusion, ACBL is an innovative and effective learning strategy for developing scientific literacy to promote professional competitiveness for medical undergraduates.

## Data Availability

The original contributions presented in the study are included in the article/Supplementary material, further inquiries can be directed to the corresponding authors.

## Footnotes

^1^ https://www.igem.org
