# Supplementary material for: Academic competition-based learning cultivates scientific literacy to promote professional competitiveness in medical undergraduates
Source: Front Public Health. 2025 Jul 3;13:1590832. doi: 10.3389/fpubh.2025.1590832 (PMC12267259; doi:10.3389/fpubh.2025.1590832)
Supplement: Supplementary file 1 [file Data_Sheet_1.docx]

**Appendix A: I-STARR Recovery Housing Research and Training Priorities Survey**

This questionnaire is being sent to Advisory Board members for the Infrastructure for Studying Treatment & Addiction Recovery Residences (I-STARR) project. We would like your input regarding the research and training topics that should be addressed by the project for people who live in recovery residences and receive medications for opioid use disorder (MOUD).

Research Priorities [ASKED OF ALL]

In this first section, we would like to know which research topics should have a higher priority compared to others. Please rate how high a priority the topic is (in your view) for research funding.

1. The development of measures to assess the capability of recovery residences to oversee self-administration of and adherence of medications for opioid use disorder (MOUD).

Low 1 2 3 4 5 High

Priority      Priority

2. Assessment of outcomes of people living in recovery housing who receive medications for opioid use disorder (MOUD) and factors that may influence outcomes

Low 1 2 3 4 5 High

Priority      Priority

3. Identification of individual, provider, and system-level barriers and facilitators to accessing recovery housing among those receiving MOUD

Low 1 2 3 4 5 High

Priority      Priority

4. The study of social networks among residents in recovery housing receiving MOUD

Low 1 2 3 4 5 High

Priority      Priority

5. Identifying strategies to overcome recovery housing operator-level barriers to supporting residents receiving MOUD

Low 1 2 3 4 5 High

Priority      Priority

6. Examining factors associated with MOUD adherence among recovery housing residents

Low 1 2 3 4 5 High

Priority      Priority

7. Assessment of recovery housing residents’ perceptions of their MOUD treatment experience

Low 1 2 3 4 5 High

Priority      Priority

8. Strategies to increase linkage between MOUD prescribers/treatment providers and recovery residence operators

Low 1 2 3 4 5 High

Priority      Priority

What other suggestions do you have for research that should be conducted in recovery housing settings?

In this next section, we would like to know which training topics should have a higher priority compared to others.

Which stakeholder group do you identify within the recovery housing field?

 Researcher

 Recovery residence operator/provider or other treatment/service provider

 Other (specify):________________________________

[SKIP LOGIC FOR PROGRAMMING: Researcher only, GO TO Researcher Training questions and end; Service provider only, GO TO Provider Training questions and end; Other or Both, answer both Researcher and Provider Training questions (NO SKIPS).]

Researcher Training Priorities

Listed below are possible topics currently under consideration for the Researcher Trainings for individuals who are interested in conducting recovery housing research. Please rate how high a priority the topic is (in your view) for including in the Researcher Training webinars.

1. Basic information on recovery housing, like different types and services provided, the history and evidence base, current research gaps, and obstacles to conducting research

Low 1 2 3 4 5 High

Priority      Priority

2. Experimental and quasi-experimental recovery housing research designs

Low 1 2 3 4 5 High

Priority      Priority

3. Sampling and collaborating with recovery housing providers/operators

Low 1 2 3 4 5 High

Priority      Priority

4. Principles and components of community-based participatory research

Low 1 2 3 4 5 High

Priority      Priority

5. Recruitment and retention of marginally housed and legal-system-involved study participants

Low 1 2 3 4 5 High

Priority      Priority

6. Treatments and medication for opioid use disorder (MOUD) and pharmacotherapies for (OUD)

Low 1 2 3 4 5 High

Priority      Priority

7. Strengths and weakness of mixed-methods designs

Low 1 2 3 4 5 High

Priority      Priority

8. Recovery housing mechanisms of action and measurement of key constructs

Low 1 2 3 4 5 High

Priority      Priority

9. Statistical methods to strengthen causal inference

Low 1 2 3 4 5 High

Priority      Priority

10. Multilevel modeling and studying “contextual” factors affecting residents

Low 1 2 3 4 5 High

Priority      Priority

11. Challenges faced by recovery housing providers/operators

Low 1 2 3 4 5 High

Priority      Priority

12. Overcoming potential challenges to moving research forward on MOUD in recovery housing

Low 1 2 3 4 5 High

Priority      Priority

What other suggestions do you have for trainings for aspiring recovery housing researchers?

Provider/Operator Trainings

Listed below are possible topics currently under consideration for training webinars for Recovery Housing Providers/Operators. Please rate how high a priority the topic is (in your view) for including in the Providers/Operators Training webinars.

1. Basic information on recovery housing, like different types and services provided, the history and evidence base, current research gaps, and obstacles to conducting research

Low 1 2 3 4 5 High

Priority      Priority

2. Components of support provided within recovery housing and how this can be delivered

Low 1 2 3 4 5 High

Priority      Priority

3. Fundamentals of recovery housing management and operations

Low 1 2 3 4 5 High

Priority      Priority

4. Resident policies regarding admission and discharge, resident rights and responsibilities, substance use screening, confidentiality and releases of information, and handling grievances

Low 1 2 3 4 5 High

Priority      Priority

5. Law/Ethics/Fiscal responsibility such as laws regarding licensing/delivery of professional services, fair housing, employment; ethics surrounding business practices and marketing; fiscal management regarding insurance, budgeting, tax requirements, and record keeping.

Low 1 2 3 4 5 High

Priority      Priority

6. Psychosocial interventions and medications for opioid use disorder (MOUD)

Low 1 2 3 4 5 High

Priority      Priority

7. Organizational-level challenges that need to be addressed when accepting residents on MOUD

Low 1 2 3 4 5 High

Priority      Priority

8. Screening applicants prescribed MOUD and other medications of concern

Low 1 2 3 4 5 High

Priority      Priority

9. MOUD diversion and risk management

Low 1 2 3 4 5 High

Priority      Priority

10. Overcoming potential tensions between prescribers and residence managers/operators

Low 1 2 3 4 5 High

Priority      Priority

11. Basic principles of research and participating in a research study

Low 1 2 3 4 5 High

Priority      Priority

12. Overcoming potential challenges to moving research forward on MOUD in recovery housing

Low 1 2 3 4 5 High

Priority      Priority

What other suggestions do you have for trainings for providers/operators of recovery housing?
